# Catalytic Performance of Post-Treated Thin-Plate ZSM-5 for Allyl Alcohol Etherification

**DOI:** 10.3390/molecules31193421

**Published:** 2026-09-25

**Authors:** Gideon Abaidoo Ocran, Huali Tan, Bosong Zhang, Jiaxing Zhang, Yi Zuo, Hong Yang, Xinwen Guo

**Affiliations:** 1 State Key Laboratory of Fine Chemicals, Frontiers Science Center for Smart Materials Oriented Chemical Engineering, School of Chemical Engineering, Dalian University of Technology, 2 Linggong Road, Dalian 116024, China; 2 School of Engineering, The University of Western Australia, Perth, WA 6009, Australia

**Keywords:** allyl alcohol, allyl ether, ZSM-5, alkali, post-treatment

## Abstract

Diallyl ether possesses unique chemical activities due to its dual functional allyl group and ether bond and is thus a high-value-added fine chemical with diverse applications. In this work, thin-plate ZSM-5 is synthesized and then subjected to alkaline post-treatment to improve its catalytic performance for allyl alcohol etherification to diallyl ether. The study shows that the post-treatment impacts on both the microstructure and acidic properties of ZSM-5. Although the selectivity of diallyl ether obtained over different ZSM-5 catalysts are similarly high, the treatment with tetrapropylammonium hydroxide and/or dilute NaOH solutions enhances the conversion of allyl alcohol. The highest turnover number of the reaction achieves when there is a moderate amount of strong acid sites in ZSM-5. This study contributes to the knowledge base for potential application of ZSM-5 in diallyl ether production from allyl alcohol.

## 1. Introduction

Diallyl ether (DAE), as a high-value-added fine chemical, possesses unique chemical activity due to its dual functional properties of allyl (-CH_2_=CH-CH_2_) and ether bond (-O-) in its molecular structure. In the conventional process, diallyl ether production involves reacting allyl halide with allyl alcohol under alkaline conditions in a solvent to achieve etherification, in what is known as the Williamson ether synthesis [1,2]. Various allylating reagents, such as allyl acetate [3] and allyl carbonate [4], have also been utilized as electrophiles to generate diallyl ether. However, the main shortcomings of these methods include the generation of halide waste, organic acid by-products [3,4,5], and wastewater, resulting in significant environmental pressure. In addition, homogeneous catalysts utilized in such process require expensive ligands in synthesis, and are difficult to reuse the spent catalyst, resulting in high industrial costs.

The development of heterogeneous catalysts, such as zeolites, can increase the reaction selectivity to >95% with reduced wastewater discharge. ZSM-5 is a typical microporous zeolite with controllable acidity and is widely used in various industrial processes. ZSM-5 has two types of intersecting 10-memebered ring channels. The straight channels (0.53 nm × 0.56 nm) align parallel to the crystallographic b-axis and the sinusoidal channels (0.51 nm × 0.55 nm) parallel to the a-axis [6,7]. Typically, the straight channels are better at promoting the migration of reactants and reducing coke formation, which is crucial for catalyst longevity [8]. The recent advancement in producing hierarchical ZSM-5 (zeolites containing both micropores and mesopores or macropores) [9,10] and nanosized ZSM-5 [11,12] provides some innovative solutions to enhance acid site accessibility and micropore channel connectivity, thereby improving catalytic activity and catalyst stability.

Our research group has the synthesis know-how on producing thin-plate ZSM-5 with short b-axis thicknesses and superior diffusion at molecular level [13]. Recently, the group applied thin-plate ZSM-5 to catalyze alkylation reaction [14] and found that it has enhanced catalytic activity and improved resistance to coke formation, owing to the short diffusion length and excellent acid site accessibility.

For allyl alcohol etherification, poor DAE selectivity over conventional ZSM-5 has been well documented. For example, Bezouhanova et al. (1998) [15] showed that allyl alcohol undergoes multiple parallel reactions including dehydration and condensation over the active sites of H-ZSM-5, leading to the formation of a complex product mixture containing diallyl ether (DAE), oxygenated compounds, hydrocarbons, and heterocyclic compounds. Anderson et al. (1995) [16] also found that DAE selectivity declines significantly as temperature increases. At 250 °C hydrocarbons became the dominant product, accompanied by a rapid catalyst deactivation due to coke deposition.

The poor performance of conventional ZSM-5 in allyl alcohol etherification is believed to originate from two aspects. Firstly, the acid strength of ZSM-5 plays a crucial role in determining the reaction pathway and product distribution. Conventional H-ZSM-5 possesses abundant weak-to-moderate acid sites, which are not sufficient to catalyze the reaction [17]. On the other side, excessive strong acid sites are more conducive to the side reactions such as coking. Secondly, the diffusion constraints imposed by the microporous structure. With a pore diameter of only 0.51–0.56 nm, the micropores of ZSM-5 are insufficient to permit the smooth diffusion of DAE, which has a molecular size (~1.02 nm) lager than that of allyl alcohol (~0.65 nm) [18]. This diffusion restriction prolongs the residence time of DAE within the micropore channels, which further intensifies side reactions and coke formation.

Considering above, we deploy the design strategy to take advantage of shortened diffusion length of thin-plate ZSM-5 and to subject it to post-treatment to tailor the acid strength to the desired moderate-to-weak range. This study investigates the impact of a range of alkali agents on the structure and acidity of thin-plate ZSM-5. It is for the first time, thin-plate ZSM-5 is used to catalyze allyl alcohol to diallyl ether under relatively mild reaction conditions. This work provides a green heterogeneous catalytic route for diallyl ether production, avoiding hazardous allyl halides, and expands the application scope of ZSM-5 zeolites to reactions involving bulky molecules.

## 2. Results and Discussion

### 2.1. Microstructure Characteristics

Figure 1 shows the XRD patterns, relative crystallinity (RC) data of ZSM-5-0 (taken as RC = 100%) and post-treated ZSM-5 samples. As seen, the five characteristic diffraction peaks of MFI topology at the 2*θ* of 7.8°, 8.8°, 23.0°, 23.9°, and 24.4° are observed in each sample, with no peaks belonging to other phase observed. This indicates that the intrinsic MFI structure of ZSM-5-0 is preserved during the post-treatment regardless of the type of alkaline treatment solution used [19,20]. Among the post-treated samples, only the two samples treated with TPAOH have a higher RC than that of ZSM-5-0, reaching the highest of 109% for ZMS-5-P. In contrast, the NaOH post-treatment results in reduced RC across all samples. Firstly, post-treatment in 0.1 mol/L NaOH results in a progressive reduction in RC with treatment time from 98% at 2 h to 93% at 15 h. When the NaOH concentration increases from 0.1 to 0.5 mol/L, RC of the post-treated samples decrease progressively from 98% to 85% after 2 h of treatment. When treated in the mixed TPAOH and NaOH (0.1 mol/L) solution for 2 h, ZSM-5-PN-2 has a RC of 105%. Alkaline post-treatment involves two competing processes: framework dissolution and recrystallization. The observation that TPAOH-treated samples exhibit RC exceeding 100% therefore indicates that the recrystallization rate exceeds the dissolution rate during the post-treatment process, relative to the reference ZSM-5-0 [21,22,23].

Figure 2 shows the SEM images of the thin-plate ZSM-5-0 and post-treated ZSM-5 samples. At the magnification shown, all samples appear to have well preserved crystal plates of smooth surfaces and sharp edges. The average b-axis thickness of the crystals is ~50–60 nm and the a-c dimensions are relatively uneven within a sample and across the samples. This seems to indicate that the post-treatment does not cause destruction or macroscopical damages to the ZSM-5 crystals.

Figure 3 shows the TEM images of the thin-plate ZSM-5 and a selection of post-treated samples. As seen in Figure 3a, the as-prepared ZSM-5-0 contains pristine thin crystals of smooth surfaces and sharp edges. After post-treatment in TPAOH solution, noticeable internal defects (~20 nm) are observed within the crystals of ZSM-5-P (Figure 3b). When post-treated in NaOH solution, the samples experience varied degrees of superficial damage to the crystals, depending on the solution concentration and treatment time. For example, in 0.1 mol/L NaOH solution, 2 h treatment results in the formation of small surface pits which seem to be randomly distributed in ZSM-5-N-0.1-2 (Figure 3c). Interestingly, extended 15 h treatment does not seem to cause obvious changes to the crystals (Figure 3d). When NaOH concentration is increased from 0.1 to 0.5 mol/L, some degree of fragmentation is observed in ZSM-5-N-0.5-2 after 2 h treatment (Figure 3e). When treated in the mixed TPAOH and NaOH solution for 2 h, small pits seem to be partially restored in ZSM-5-PN-2 (Figure 3f).

Figure 4 presents the N_2_ adsorption–desorption isotherms of the thin-plate ZSM-5-0 and post-treated ZSM-5 samples. As shown in the figure, all samples display type I physisorption behaviour typical of a microporous zeolite. In addition, a minor hysteresis loop is observed at P/P_0_ = 0.9–1.0 for each sample, indicating the presence of a small amount of mesopores.

Table 1 summarizes the texture properties of the samples extracted from the isotherms, including DFT calculated surface area (S_DFT_), micropore area and volume (S_micro_, V_micro_), and mesopore area and volume (S_meso_, V_meso_).

As shown in Table 1, the TPAOH-treated ZSM-5-P sample exhibits slightly higher S_DFT_, S_meso_ and V_total_ than the parent ZSM-5-0. In contrast, all NaOH-treated samples show a decrease in S_DFT_ to varying degrees compared to ZSM-5-0. Among the samples treated with the same NaOH concentration for different time (ZSM-5-N-0.1-x), both S_DFT_ and S_micro_ increase progressively with the prolonging treatment time. For the samples treated with varying NaOH concentrations for the same time (ZSM-5-N-x-2), S_DFT_ and S_micro_ also increase significantly with increasing concentration. For the combined TPAOH + NaOH treatment (ZSM-5-PN-2), the textural properties are largely preserved and remain comparable to that of ZSM-5-0. The above results show that firstly, TPAOH can effectively suppresses excessive framework dissolution during post-treatment of ZSM-5. It has unique ability to induce internal cavities with ZSM-5 crystals while preserving external geometries of crystals. Secondly, the NaOH treatment would initially dissolve the microporous framework structure, resulting in decreased S_DFT_ and S_micro_ comparing to that of ZSM-5-0. As the treatment proceeds, surface pits and defects continuously form (Figure 3d), leading to a partial recovery of S_DFT_ and S_micro_ in the treated samples.

Table 1 also lists the actual Si/Al ratio determined through ICP-OES of the samples. Firstly, ZSM-5-0 has an actual Si/Al ratio of 56, slightly higher than the nominal ratio of 50. The post-treatment consistently results in a reduction in Si/Al ratio for all post-treated samples, indicating a net desilication of samples after the alkaline treatment. Notably, TPAOH and TPAOH + NaOH treated samples both show less reduction in Si/Al ratios than those treated in pure NaOH solutions. This indicates that pure NaOH solution causes more severe desilication to ZSM-5 than TPAOH or TPAOH-containing solution. Such effect is not likely caused by the slight pH differences in the treatment solutions as listed in Table 1.

### 2.2. Acidity Properties

Figure 5 shows the NH_3_-TPD profiles of the thin-plate ZSM-5-0 and post-treated ZSM-5 samples. As seen, each sample displays a NH_3_ desorption curve (solid black line) that can be deconvoluted to three desorption peaks in temperature ranges of 475–525 K, 550–620 K, and 540–710 K. They represent the weak (orange dashed line), medium (purple dashed line), and strong (pink dashed line) acidic sites in ZSM-5, respectively [14,24].

Table 2 lists the relative amounts of the three types of acid sites in each sample obtained by Gaussian peak fitting of its NH_3_-TPD curve. As seen, compared to the parent ZSM-5-0, the total acid amount decreases across all post-treated samples, except for ZSM-5-N-0.5-2. For TPAOH-treated ZSM-5-P, while the amounts weak and medium-strength acid sites remain largely unchanged, the strong acid amount decreases noticeably, so does the total acid amount. On the other hand, depending on the concentration and/or treatment time, NaOH solution has different impact to the acidity of post-treated ZSM-5. For ZSM-5-N-0.1-2, all acid types decrease sharply relative to that of ZSM-5-0, particularly the strong acid amount drops from 145 to 83 μmol/g_cat_ (~43% reduction) and weak acid amount declines from 96 to 59 μmol/g_cat_ (~39% reduction). This results in ~37% reduction of the total acid amount in ZSM-5-N-0.1-2 compared to ZSM-5-0. Interestingly, prolonged treatment to 15 h in the same 0.1 mol/L NaOH solution has seen a strong recovery in amounts of weak, strong and total acid in ZSM-5-N-0.1-15. In fact, the medium-strength acid in this sample exceeds that in ZSM-5-0. Furthermore, pots-treatment in high concentration of NaOH solution has resulted in a significant increase in both the weak acid and total acid amounts in ZSM-5-N-0.5-2, while maintaining the amounts of medium-strength and strong acids compared to ZSM-5-0 [25,26]. Finally, the acidity characteristic of ZSM-5-PN-2 (treated in TPAOH + NaOH (0.1 mol/L) for 2 h) appears more impacted by the NaOH than TPAOH in the treatment solution.

To identify the nature of the active sites and quantify the acid site distribution, pyridine-adsorbed FTIR (Py-IR) spectroscopy analysis was performed. As shown in Figure 6a, the bands at ~1540 cm^−1^ and ~1450 cm^−1^ are assigned to pyridinium ions bound to Brønsted acid sites (BASs) and pyridine coordinated to Lewis acid sites (LASs), respectively. The initial reaction rates X(AA) are then plotted against the amounts of BASs (Figure 6b) and amounts of LAS (Figure 6c) in different ZSM-5 series samples. Clearly, the results indicate that there is no obvious relationship between them. This is because the water from dehydration of allyl alcohol may lead to the transformation of Lewis acid sites to Bronsted acid sites. Furthermore, the conversion is not only related to the amount of acid, but also to the diffusion rate of molecules in the pore channels. Therefore, we did not discuss the influence of specific acid site on this reaction, but that of acid amount on the catalytic activity.

### 2.3. Catalytic Allyl Alcohol Etherification to Diallyl Ether over ZSM-5

Figure 7 presents the allyl alcohol etherification results over ZSM-5.

As seen in Figure 7a, the conversion of allyl alcohol (X(AA)) over ZSM-5-0 is 6.3% during 12 h time-on-stream (TOS). Over TPAOH-treated ZSM-5-P, X(AA) improves obviously to 13.6%. Over ZSM-5 catalysts treated with NaOH, X(AA) reaches the highest (14.5%) over ZSM-5-N-0.1-2 (the catalyst treated in 0.1 mol/L solution for 2 h) but remains significantly lower over all other NaOH-treated ZSM-5. X(AA) over mixed TPAOH + NaOH post-treated ZSM-5-PN-2 is lower than either of ZSM-5-P or ZSM-5-N-0.1-2. On the other hand, as also seen in Figure 7a, greater than 99% selectivity of diallyl ether (S(DAE)) is achieved over all ZSM-5 catalysts including ZSM-5-0. In contrast, the ZSM-5-C sample, which is not the thin-plate morphology, delivers a much lower X(AA) (~3.1%) and S(DAE) (20%) under identical reaction conditions, confirming the diffusion advantage of having thin-plate ZSM-5 crystals.

Figure 7b presents the relationship between the turnover number (TON) and amount of strong acid in ZSM-5 catalysts. TON value quantifies the number of reactant molecules that a single active site of the catalyst can convert into products in 12 h. It is seen that the TON exhibits a general correlation to the strong acid amount. In other words, a proper strong acid amount (70–100 μmol/g_cat_) gains a high TON. This is consistent with the understanding that etherification is catalyzed by strong acid sites, but excessive strong acid sites would promote coking of products and causing deactivation of the catalyst.

Figure 8 shows the TGA curves of the spent catalysts measured in air. There are three weight loss events revealed by the TGA curve of each sample. Firstly, the weight loss event observed below 150 °C is associated with the removal of physically adsorbed moisture and light hydrocarbon products. The second weight loss event is observed between 150 and 350 °C and it is due to the decomposition of coke species [27]. The final weight loss event above 350 °C is assigned to the combustion of coke species [28,29]. The coke content (measured as the sum of both 2nd and 3rd weight loss events) on the spend catalysts are also listed in the figure.

As seen in Figure 7a, three catalysts of ZSM-5-N-0.1-2, ZSM-5-P, and ZSM-5-PN-2 deliver the highest X(AA) among all catalysts studied, they also show similar levels of coking. However, ZSM-5-N-0.1-2 stands out with the highest TON, due to its relatively suitable acid property, which promotes the etherification and, at the same time, resists secondary reactions feeding into coking.

## 3. Experimental Section

### 3.1. Chemicals Used in Synthesis

The synthesis and post-treatment of the thin-plate ZSM-5 zeolite consumes the following chemicals reagent: tetrapropylammonium bromide (TPABr, >98%, Shandong Hengtai Chemical Co., Ltd., Heze, China), colloidal silica (30 wt.%, Shanghai Macklin Biochemical Co., Ltd., Shanghai, China), aluminum sulfate hydrate (Al_2_(SO_4_)_3_·18H_2_O, >99%, Tianjin Guangfu Technology Development Co., Ltd., Tianjin, China), urea (NH_2_CONH_2_, >99%, Tianjin Damao Chemical Reagent Factory, Tianjin, China), ethylamine (CH_3_CH_2_NH_2_, 68–72 wt% in H_2_O, Shanghai Aladdin Biochemical Technology Co., Ltd., Shanghai, China), tetrapropylammonium hydroxide (TPAOH, 25 wt% in water, Shanghai Aladdin Biochemical Co., Ltd., Shanghai, China), ammonium nitrate (NH_4_NO_3_, >99%) prepared from nitric acid (HNO_3_, 65 wt%, Beijing Chemical Reagent Co., Ltd., Beijing, China) and ammonia solution (NH_4_OH, 25%, Tianjin Damao Chemical Reagent Factory, Tianjin, China), gamma alumina (γ-Al_2_O_3_, Qingdao Marine Chemical Co., Ltd., Qingdao, China) and Sesbania powder (98 wt%, Hangzhou Zhongtuan Biotechnology Co., Ltd., Hangzhou, China).

### 3.2. Synthesis and Preparation of ZSM-5 Catalysts

Synthesis of thin-plate ZSM-5: Thin-plate ZSM-5 (nominal Si/Al = 50) was synthesized with in a semi-industrial scale 20 L stainless steel reactor, following the seed-induced strategy reported in our previous works [13,30], using a gel composition of 1 SiO_2_: 0.3 CH_3_CH_2_NH_2_: 0.1 TPABr: 0.02 Al_2_O_3_: 30 H_2_O: 0.15 CO(NH_2_)_2_: 5 wt.% seeds (respect to the silica in TEOS). Typically, the deionized water, TPABr, ethylamine, colloidal silica (30 wt.%), and Silicalite-1 seed suspension were mixed and stirred. Then, Al_2_(SO_4_)_3_·18H_2_O and CO(NH_2_)_2_ were dissolved in deionized water, respectively, and added to the mixture to obtain a synthesis gel. The gel was transferred into the 20 L stainless-steel reactor and heated at 170 °C for 24 h. The solid product was repeatedly filtered and washed with deionized water until neutral, dried at 80 °C for 12 h, and calcined at 540 °C in air for 6 h. The resulting powder is named ZSM-5-0. For comparison, a ZSM-5 particle sample (denoted as ZSM-5-C) was synthesized following the same procedure as ZSM-5-0 but without the addition of CO(NH_2_)_2_.

Post-treatment of ZSM-5 in TPAOH solutions: The as-prepared thin-plate ZSM-5 powder was post-treated with organic hydroxide TPAOH. Typically, 8.0 g of ZSM-5 powder was dispersed and mixed well in 80 mL of TPAOH solution (0.06 mol/L). The mixture was transferred to a Teflon-lined vessel stainless-steel autoclave and heated to and maintained at 170 °C for 48 h. After being cooled down, pH of the treatment solution was measured and recorded before the solid product was recovered by centrifugation. It was then washed with deionized water until the pH of the washing water was neutral, dried at 80 °C for 12 h, and calcined at 540 °C in air for 4 h to obtain the TPAOH-treated ZSM-5 catalysts, denoted as ZSM-5-P.

Post-treatment of ZSM-5 in NaOH solution: The as-prepared thin-plate ZSM-5 powder was also post-treated in NaOH solutions of different concentrations for different times. In one group, 1.0 g of ZSM-5 powder was treated in 15 mL of NaOH solution (0.1 mol/L) for 2, 5 or 15 h under vigorous stirring at 50 °C. In another, 1.0 g of ZSM-5 powder was treated in 15 mL of NaOH solution of molar concentration of 0.1, 0.2 or 0.5 mol/L for 2 h under vigorous stirring at 50 °C. For each treatment, pH of the solution was measured and recorded at the end of each treatment. After the treatment, the solid was filtered, washed with deionized water until the pH of the washing water was neutral, dried at 80 °C for 12 h, and calcined at 540 °C in air for 4 h to obtain the NaOH-treated ZSM-5 catalysts, denoted as ZSM-5-N-*C*-*t*. Here, N refers NaOH, *C* refers to the molar concentration of NaOH solution, and *t* refers to the time of treatment in hours.

Post-treatment of ZSM-5 in mixed TPAOH and NaOH solution: Finally, 1.0 g of as-prepared thin-plate ZSM-5 powder was post-treated in 15 mL of mixed solution of NaOH (0.1 mol/L, 11.25 mL) and TPAOH (1.23 mol/L, 3.75 mL) for 2 h, under otherwise identical procedures as in other NaOH post-treatments. The resulting sample is denoted as ZSM-5-PN-2.

Preparation of ZSM-5 extrudate catalyst: In this study, the as-prepared and post-treated thin-plate ZSM-5 powders was extruded and then ion-exchanged for the catalytic performance evaluation as catalyst. As an example, the extrudate of the as-prepared thin-plate ZSM-5 was prepared with the following procedure. First, 6.0 g of thin-plate ZSM-5 powder, 1.5 g of γ-Al_2_O_3_ powder, and 1.0 g of Sesbania powder were premixed, followed by the addition of 5.5 mL of HNO_3_ (10% *v*/*v*). The resulting mixture was subsequently shaped into cylindrical rods of 2.0 mm diameter by rapid extrusion moulding, dried at room temperature for 12 h, dried at 80 °C for 7 h, and then calcined at 540 °C in air for 4 h. The resulting extruded ZSM-5 was finally cut into 2 mm × 2 mm pellets.

### 3.3. Characterization

Powder X-ray diffraction (XRD) patterns were obtained on a Rigaku SmartLab 9 diffactometer (Tokyo, Japan), which was operated with Cu Kα radiation (λ = 1.5418 Å) with a scanning range of 2*θ* = 5–50°, scanning speed of 2° min^−1^, scanning step of 0.02° at 40 kV and 100 mA. The relative crystallinity (RC) was calculated as the ratio of the total intensity of the five characteristic peaks of the MFI topology of each sample to that of the ZSM-5-0. The overall elemental compositions of the samples were determined using an inductively coupled plasma-optical emission spectrometer (ICP-OES) on a PerkinElmer AVIO 500 optical emission spectrometer (Waltham, Massachusetts, US). The acidity of the samples was obtained through NH_3_ temperature-programmed desorption (NH_3_-TPD) on a ChemBET Pulsar TPR/TPD automatic chemisorption instrument (Quantachrome, Boynton Beach, FL, USA). The desorption of NH_3_ was performed by ramping up the temperature from 400 K to 923 K at a heating rate of 10 K per minute. The signal was collected by using a TCD as the detector. Scanning electron microscopy (SEM) images were collected on a Hitachi (Tokyo, Japan) SU8220 at an acceleration voltage of 5 kV to observe the morphology and crystal sizes. Transmission electron microscopy (TEM) images were taken using a JEM-F200 instrument (Jeol Company, Tokyo, Japan) at an acceleration voltage of 200 kV. Nitrogen adsorption–desorption isotherms were measured with an Autosorb SI gas adsorption analyzer (Quantachrome, Boynton Beach, FL, USA). Before measurements, about 0.05 g of the sample was heated at 300 °C for 8 h under vacuum. The specific surface area was determined by the density functional theory (DFT) method, assuming a cylindrical pore geometry with the N_2_@77K zeolite/silica kernel. The micropore area and volume were obtained via the t-plot method. The total pore volume was taken as the value at a relative pressure of 0.95. Thermogravimetric (TGA) measurements were conducted using an SDTQ-600 analyzer (TA Instruments Company, New Castle, DE, USA) to analyze the spent ZSM-5 catalysts. Pyridine adsorption FT-IR spectroscopy (Py-IR) was measured using a Bruker (Billerica, MA, USA) EQUIOX-55 Fourier transform infrared spectrometer. After pretreatment under vacuum at 623 K for 30 min, the sample was cooled to room temperature to record the background spectrum, and then, pyridine molecules were introduced for 15 min. Subsequently, the samples were evacuated at 473 K and 723 K for 35 min to remove pyridine molecules, and the results were obtained using an infrared spectrometer at room temperature.

### 3.4. Catalytic Evaluation

The etherification reaction of allyl alcohol was carried out in a fixed-bed reactor. In a typical reaction test, 4.0 g of extrudate catalyst was placed in the middle zone of a stainless-steel tube reactor with an inner diameter of 13 mm and packed with carborundum (SiC) balls below and above a quartz wool layer, with the catalyst between the layers of quartz wool. The system pressure of 1.0 MPa was maintained using nitrogen. The weight hourly space velocity (WHSV) of allyl alcohol was set at 0.8 h^−1^. The flow rate of allyl alcohol was 3.7 mL/h and was fed to the reactor by a controlled pump. The reaction was then carried out at 393 K. The product effluent from the tube reactor was cooled in a storage tank placed inside a refrigerator. The sampling was done at a time interval of 4 h. The products of the reaction were analyzed on an Agilent 7890F gas chromatograph with a flame ionisable detector (FID) and a capillary column (30 m × 0.25 mm × 0.5 µm). All catalytic performance data were obtained from at least three independent runs under identical conditions, and the reported values are the averages taken during the stable period of catalyst performance. The etherification main reaction follows the stoichiometry: 2AA → 1DAE + H_2_O. Diallyl ether (DAE) was the main product. The conversion of allyl alcohol (X(AA)), selectivity of diallyl ether (S(DAE)), and turnover number (TON) were calculated with Equations (1)–(3), respectively.

*X*(AA) = [*n*(AA)_in_ − *n*(AA)_out_]/*n*(AA)_in_ (1)

*S*(DAE) = 2 × *n*(DAE)/[*n*(AA)_in_ − *n*(AA)_out_] (2)

*TON* = *n*(AA)_in_ × *X*(AA)/[*m*_cat_ × *c_strong_*] (3)

Here, *n*(AA)_in_ and *n*(AA)_out_ stand for the inlet and outlet molar numbers of AA, respectively. *n*(DAE) is the molar number of DAE, *m*_cat_ is the weight of ZSM-5 catalyst, and *w*_Al_ is the weight percent of Al in the corresponding catalyst, which is determined using an ICP-OES.

## 4. Conclusions

This work reports a post-treatment strategy to tune the acidity of thin-plate ZSM-5 catalysts for allyl alcohol etherification to diallyl ether, thus taking the advantage of the short-diffusion length inherent of the thin-plate ZSM-5. The study examines the effects of TPAOH and/or NaOH solutions on the microstructure and acidity of ZSM-5, as well as impacts to their catalytic activities in allyl alcohol etherification to diallyl ether. Specifically, the study has achieved the following.

Firstly, the MFI structure is well preserved after all post-treatments. TPAOH treatment creates internal hollow structure to ZSM-5 crystals, increases mesopores area and relative crystallinity. NaOH treatment causes some degree of framework dissolution and surface pitting, even crystal fragmentation at high concentration of treatment solution. The combined TPAOH + NaOH treatment suppresses framework dissolution.

Secondly, TPAOH treatment selectively reduces strong acid sites while preserving weak and medium-strength acid amounts. NaOH treatment at low concentration of 0.1 mol/L initially reduces all types of acid sites, but prolonged treatment time partially restores them. NaOH treatment at high concentration of 0.5 mol/L increases all types of acid sites. The combined TPAOH + NaOH (0.1 mol/L) treatment increases the weak acid sites but reduces strong acid sites significantly.

Finally, greater than 99% selectivity toward diallyl ether is achieved over all catalysts. A general trend is observed between the turnover number (TON) and strong acid amount in ZSM-5, revealing that the optimum strong acid amount is 70–100 μmol/g_cat_ for the highest TON. Excessive strong acid sites promote coking and inhibit the main reaction. Among all catalysts, ZSM-5-N-0.1-2 exhibits the best performance due to its suitable acidity. Comparative experiments confirm that the thin-plate morphology provides a significant diffusion advantage, which is essential for overcoming the diffusion constraints of the microporous structure. Overall, this study demonstrates that combining thin-plate morphology with appropriate acidity tuning offers an effective strategy for designing zeolite catalysts for the green production of diallyl ether, broadening the potential applications of ZSM-5 in fine chemical synthesis.

## Figures and Tables

**Figure 1:**
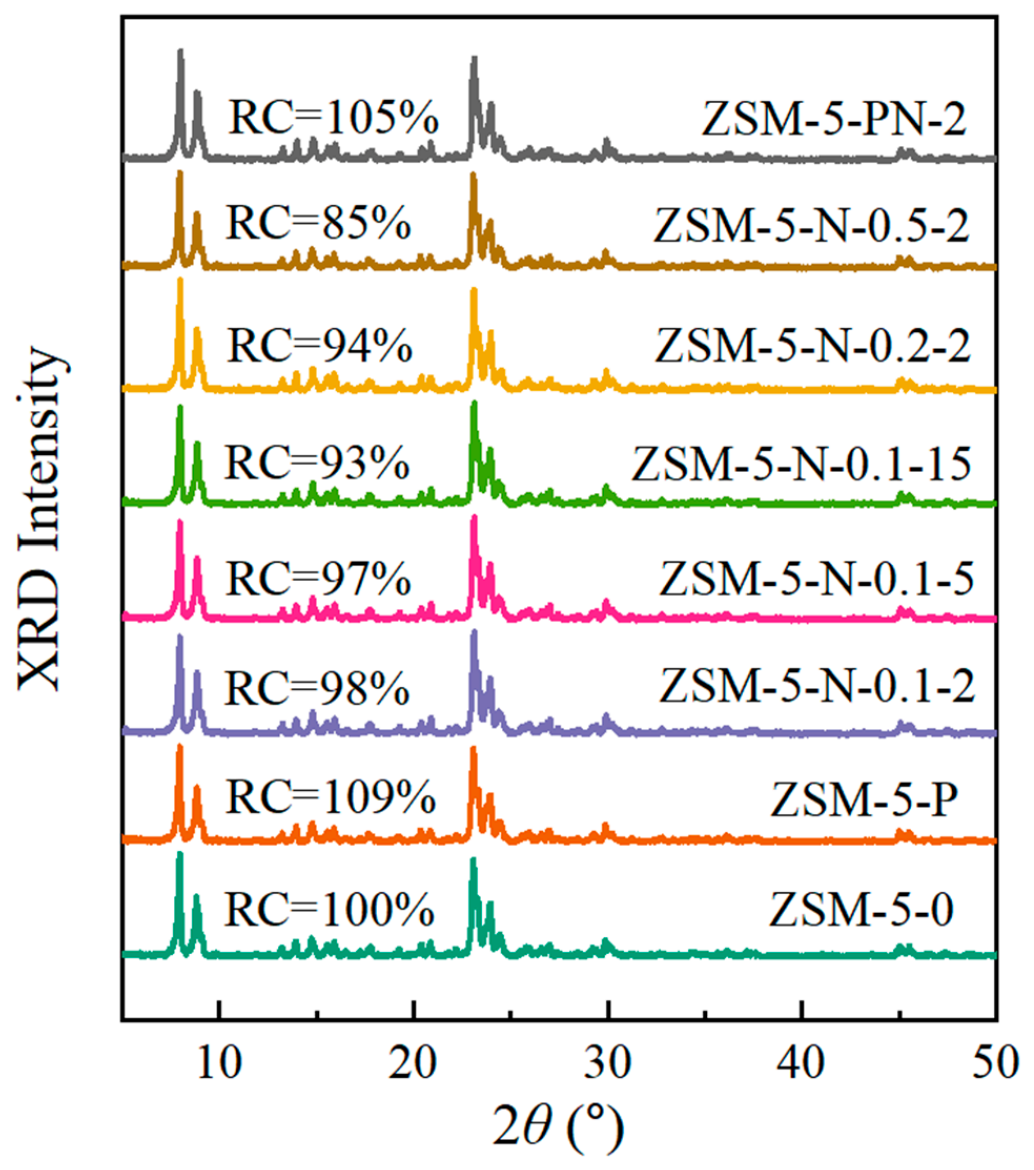
XRD patterns of thin-plate ZSM-5-0 and post-treated ZSM-5 samples with different alkaline solutions.

**Figure 2:**
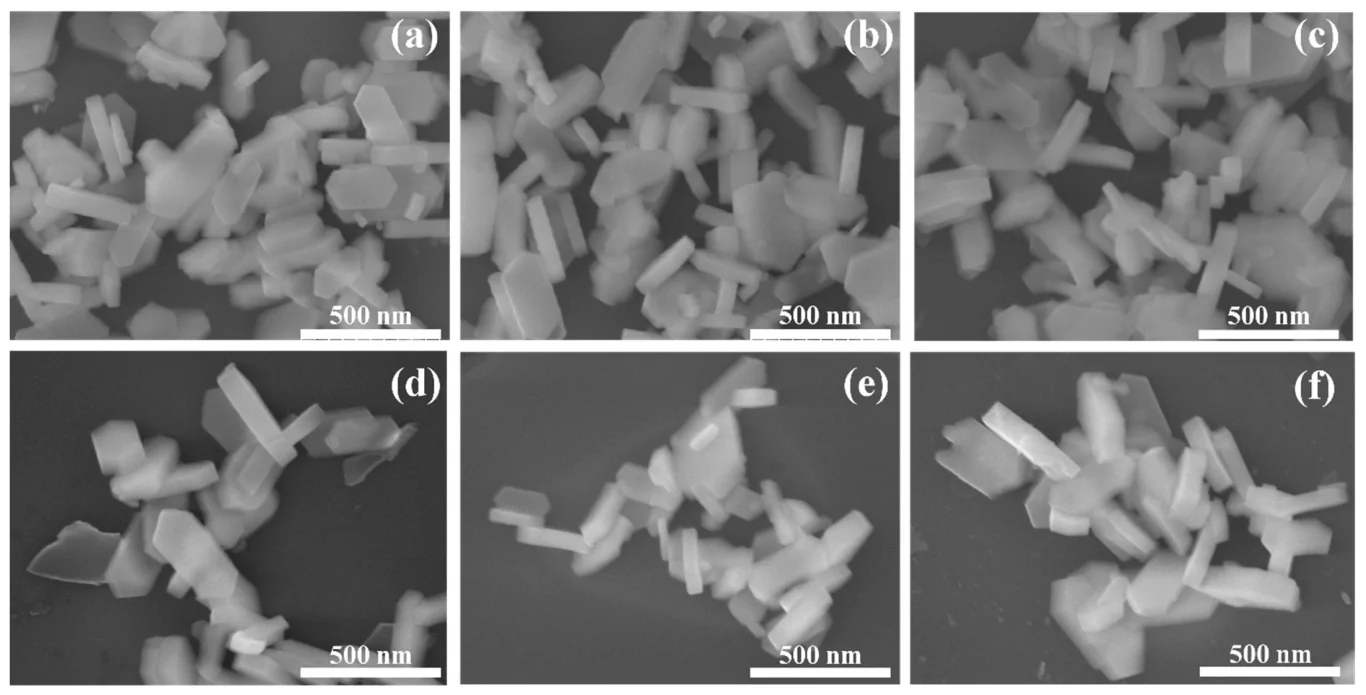
SEM images of thin-plate ZSM-5-0 and post-treated ZSM-5 samples with different alkaline solutions. (**a**) ZSM-5-0, (**b**) ZSM-5-P, (**c**) ZSM-5-N-0.1-2, (**d**) ZSM-5-N-0.1-15, (**e**) ZSM-5-N-0.5-2 and (**f**) ZSM-5-PN-2.

**Figure 3:**
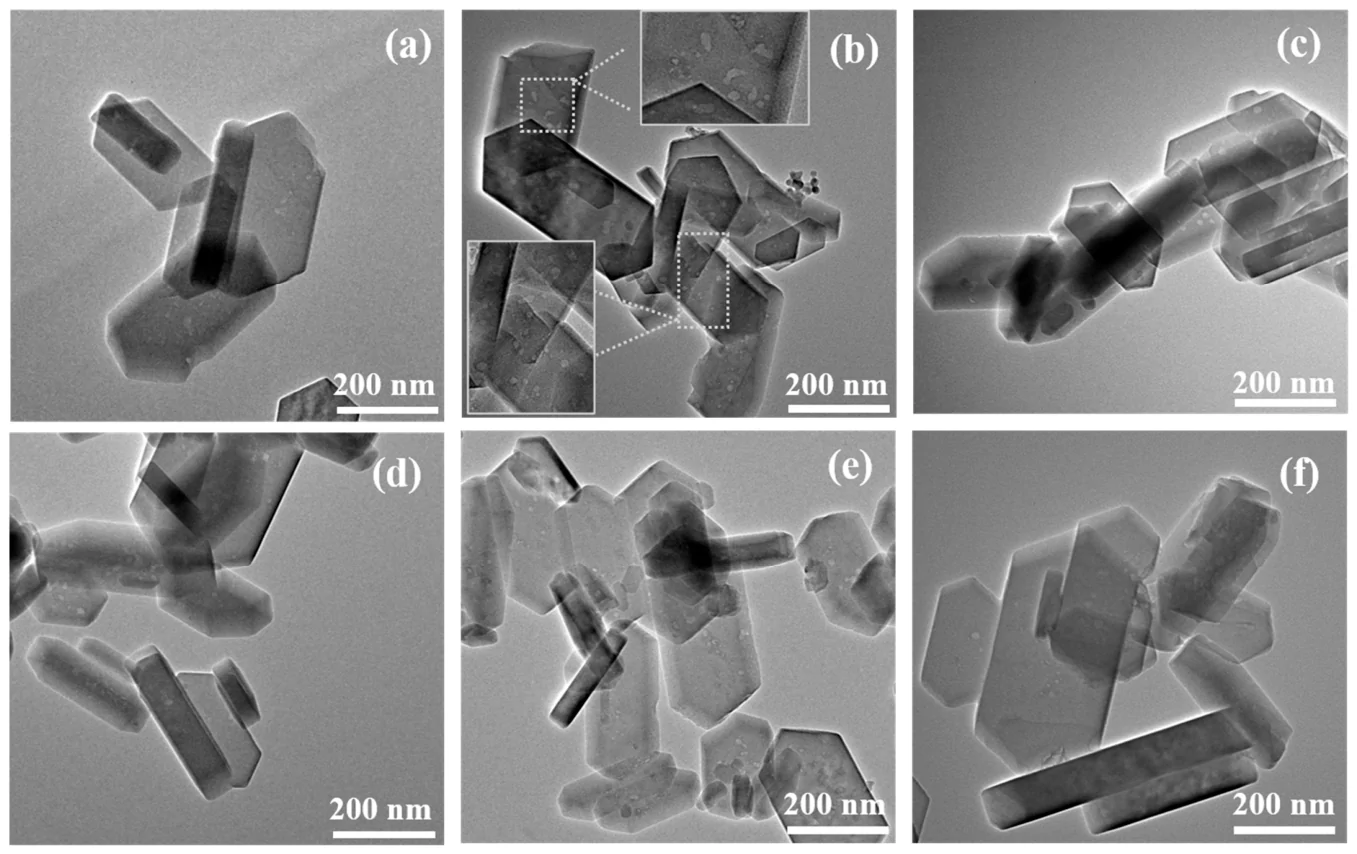
TEM images of thin-plate ZSM-5-0 and post-treated ZSM-5 samples: (**a**) ZSM-5-0, (**b**) ZSM-5-P, (**c**) ZSM-5-N-0.1-2, (**d**) ZSM-5-N-0.1-15, (**e**) ZSM-5-N-0.5-2, and (**f**) ZSM-5-PN-2.

**Figure 4:**
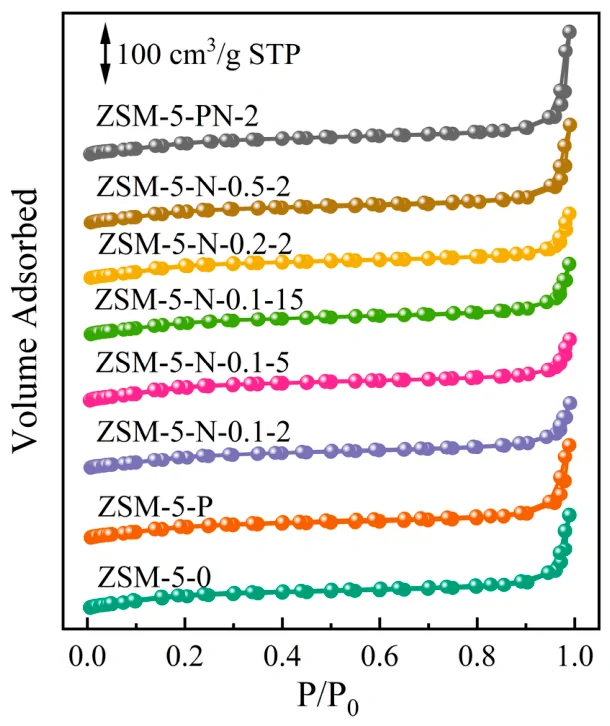
N_2_ adsorption–desorption isotherms of thin-plate ZSM-5-0 and post-treated ZSM-5 samples. The scale of the vertical coordinate (100 cm^3^/g) is shown in the figure.

**Figure 5:**
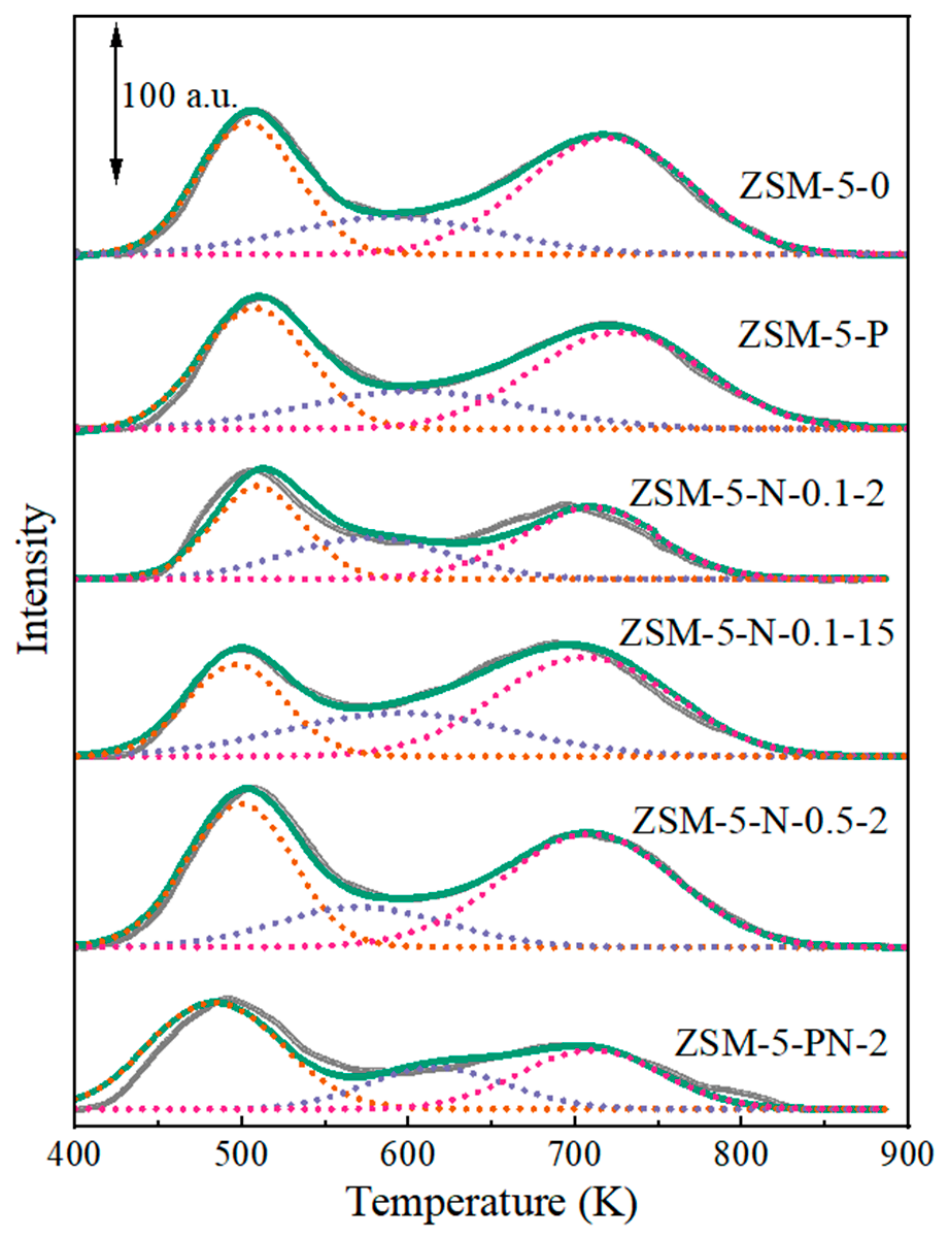
NH_3_-TPD profiles and deconvoluted NH_3_ desorption peaks of thin-plate ZSM-5-0 and post-treated ZSM-5. The scale of the vertical coordinate (100 a.u.) is shown in the figure.

**Figure 6:**
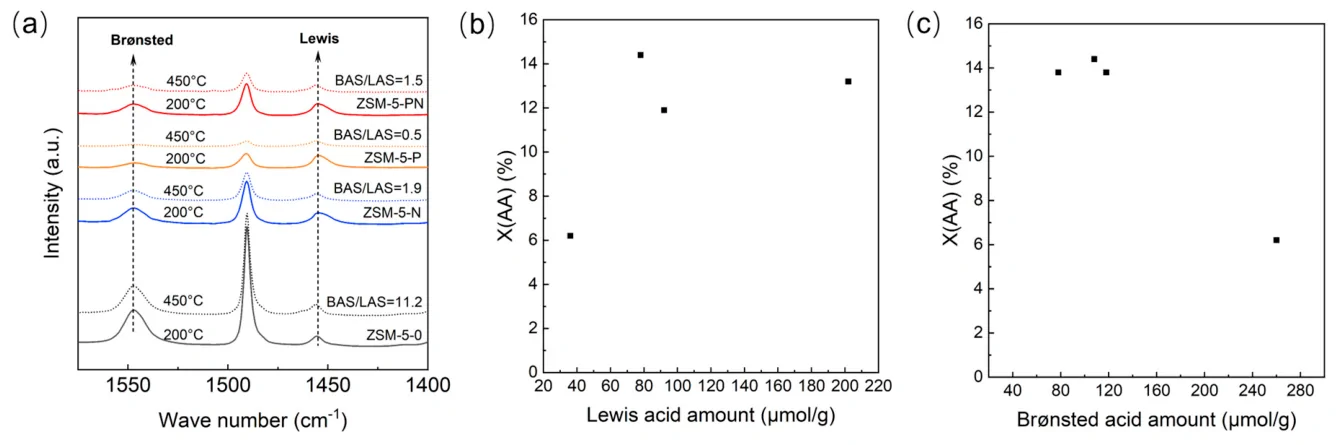
(**a**) Py-IR spectra of ZSM-5 nanosheet precursor and alkali treated samples. The correlation with (**b**) Lewis acid and (**c**) Brønsted acid between allyl alcohol conversion rate.

**Figure 7:**
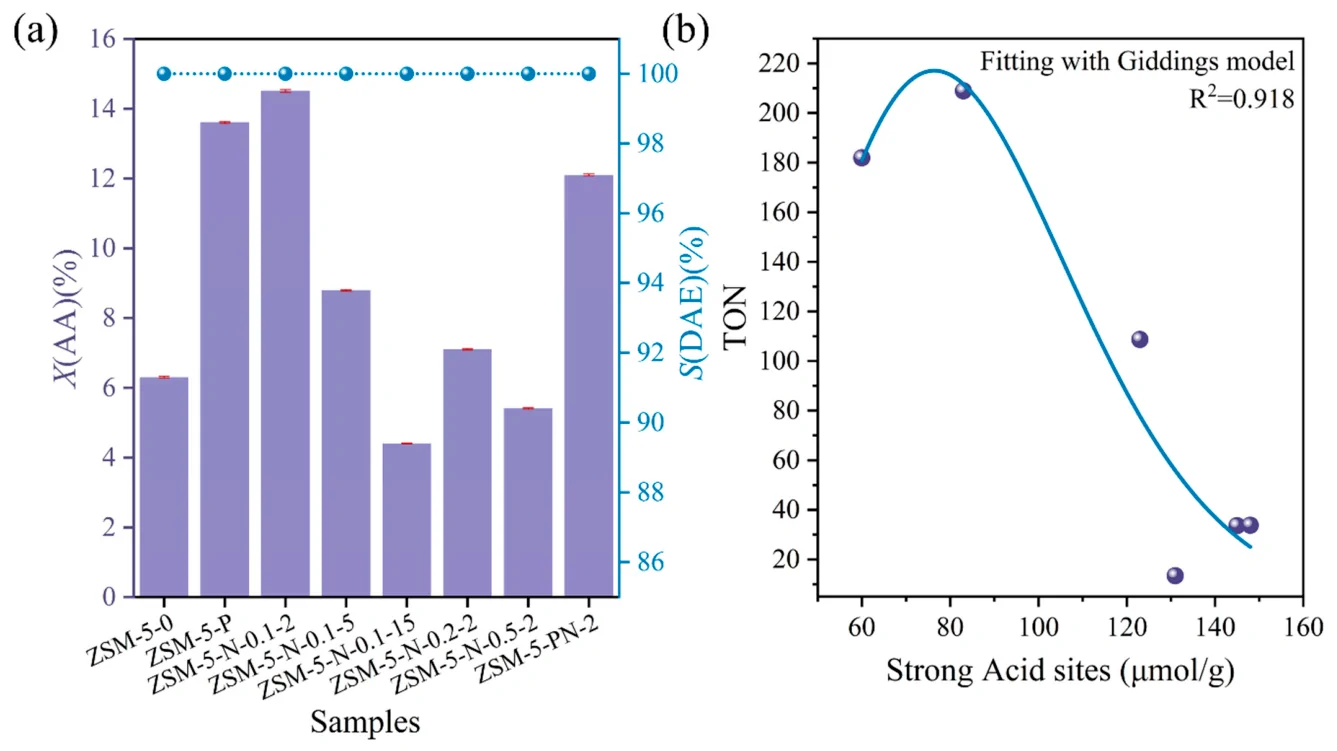
Catalytic performance of allyl alcohol etherification over ZSM-5-0 and post-treated ZSM-5 catalysts. (**a**) Conversion of allyl alcohol (bar graph) and selectivity of diallyl ether (point plot). (**b**) Plots of TON versus strong acid amount. Reaction conditions: 393 K, 1.0 MPa, cat. 4.0 g, and WHSV of allyl alcohol 0.8 h^−1^.

**Figure 8:**
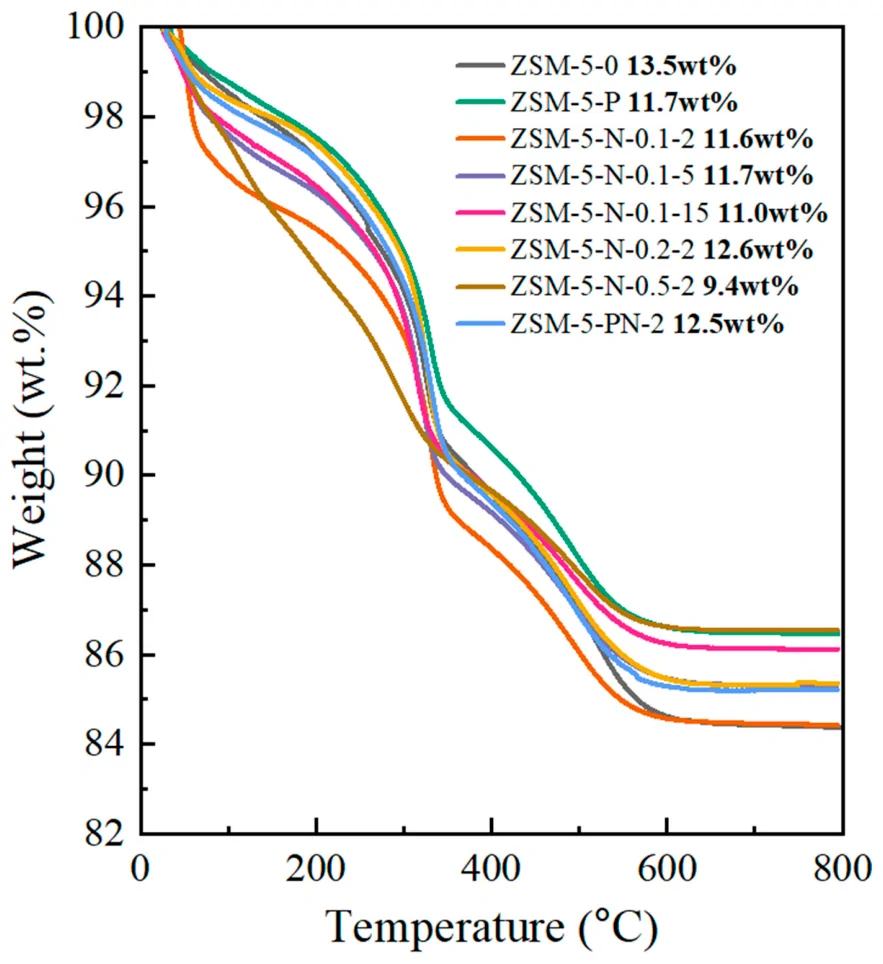
TGA curves of the spent thin-plate ZSM-5-0 and post-treated ZSM-5 samples after allyl alcohol etherification reaction (TOS = 12 h).

**Table 1:** Texture properties, actual Si/Al molar ratio, and pH values of post-treatment solution of thin-plate ZSM-5-0 and post-treated ZSM-5 samples.

Samples	pH	^a^ Si/Al	^b^ RC (%)	Surface Area (m^2^/g)			Pore Volume (cm^3^/g)	
				^c^ S_DFT_	^d^ S_micro_	^e^ S_meso_	^d^ V_micro_	^f^ V_total_
ZSM-5-0	/	56	100	379	337	40	0.15	0.23
ZSM-5-P	12.7	53	109	404	361	43	0.16	0.24
ZSM-5-N-0.1-2	13.0	46	98	355	310	45	0.14	0.22
ZSM-5-N-0.1-5	13.0	/	97	368	329	39	0.14	0.22
ZSM-5-N-0.1-15	13.0	47	93	371	333	38	0.15	0.23
ZSM-5-N-0.2-2	13.0	/	94	373	335	38	0.16	0.23
ZSM-5-N-0.5-2	13.7	45	85	374	336	38	0.16	0.23
ZSM-5-PN-2	13.4	53	105	376	340	36	0.14	0.23

^a^ molar ratio of Si/Al determined using ICP-OES. ^b^ RC determined by XRD. ^c^ S_DFT_ obtained through the DFT method. ^d^ S_micro_ and V_micro_ determined using the t-plot method. ^e^ S_meso_ = S_DFT_ − S_micro_. ^f^ V_total_ is the specific adsorption volume at P/P_0_ = 0.95.

**Table 2:** Acidity properties of thin-plate ZSM-5-0 and post-treated ZSM-5 samples determined by NH_3_-TPD measurements.

Samples	Acid Amount (μmol/g_cat_)			
	Total	c_weak_ (T_1_, K)	c_medium_ (T_2_, K)	c_strong_ (T_3_, K)
ZSM-5-0	298	96 (506)	57 (590)	145 (718)
ZSM-5-P	282	96 (511)	62 (604)	123 (713)
ZSM-5-N-0.1-2	189	59 (503)	47 (564)	83 (696)
ZSM-5-N-0.1-15	269	65 (498)	73 (591)	131 (700)
ZSM-5-N-0.5-2	314	114 (503)	51 (574)	148 (709)
ZSM-5-PN-2	212	109 (496)	43 (629)	60 (718)

T_1_, T_2_ and T_3_ denote the peak temperatures of NH_3_ desorption peaks from low to high temperatures, respectively.

## Data Availability

The original contributions presented in this study are included in the article.
